# Two sides of the coin: Feedback-driven landscape formation results in trade-off between establishment and resilience of marram grass

**DOI:** 10.1007/s00442-025-05693-5

**Published:** 2025-04-05

**Authors:** Solveig Höfer, Lissie de Groot, Nathan Scanlan, Eva Lansu, Max Rietkerk, Martin Wassen, Tjisse van der Heide, Valérie C. Reijers

**Affiliations:** 1 Conservation Ecology Group, Groningen Institute for Evolutionary Life Sciences, University of Groningen, 9700 AA Groningen, The Netherlands; 2 Department of Coastal Systems, Royal Netherlands Institute for Sea Research, 1790 AB Den Burg, The Netherlands; 3 Department of Environmental System Science, ETH Zürich, Universitätstrasse 2, 8092 Zurich, Switzerland; 4 Department of Physical Geography, Faculty of Geosciences, Utrecht University, 3508 TC Utrecht, The Netherlands; 5 Copernicus Institute of Sustainable Development, Environmental Sciences Group, Utrecht University, Utrecht, The Netherlands

**Keywords:** Biogeomorphic gradient, *Calamagrostis arenaria*, Plant life-stages, Habitat-modifying plants, Coastal dunes

## Abstract

Habitat-modifying plants engineer biogeomorphic landscapes through self-reinforcing interactions with their physical environment, or so-called ‘biogeomorphic feedbacks’. Nevertheless, benefits can vary across a biogeomorphic landscape gradient and between plant-life stages. For instance, European marram grass forms dunes by trapping sediments which triggers plant growth, in turn promoting sediment trapping. Yet, by increasing dune height and vegetation cover, marram grass mitigates sediment dynamics, inhibiting sediment-growth feedbacks, which ultimately leads to its demise. However, little is known about how dune formation affects the growth and survival of marram grass at different life stages. Therefore, we performed a two-level field experiment testing the effect of position on marram grass across the biogeomorphological dune gradient (beach, foredune, backdune) on (i) the establishment success of juvenile transplants and (ii) the resilience of mature plants to disturbance by above-ground biomass removal, over one growing season. Although juvenile transplants grew similarly well across the dune gradient, significantly fewer beach transplants (67%) survived compared to the foredune- and backdune transplants. Conversely, survival of mature disturbed marram grass (100%) was unaffected, yet recovery was highest at the beach and significantly decreased across the dune gradient. We could link these opposing responses to habitat modification. In heavily modified dune habitats sediment stabilization aided juvenile establishment, whereas the high sediment dynamics of unmodified beaches facilitated adult resilience indicating dune formation invokes a trade-off between establishment and resilience. Our findings highlight the importance of assessing life stage-dependent differences in environmental requirements of habitat-modifying plants to understand population dynamics and landscape-forming processes.

**Supplementary Information:**

The online version contains supplementary material available at 10.1007/s00442-025-05693-5.

## Introduction

Habitat-modifying plants are considered ‘ecosystem engineers’ (Jones et al. 1994, 1997) because they engineer their own habitat by interacting with their physical environment (Jones et al. 1994, 1997; Corenblit et al. 2007, 2015). These plant-environment interactions, or ‘biogeomorphic feedback loops’, underlie the formation of vegetated coastal landscapes worldwide (Orth et al. 2006; Corenblit et al. 2007; Martinez and Psuty 2008; Friess et al. 2012). Emerging seagrass meadows, salt marshes, mangrove forests, and coastal dunes harbor diverse species assemblages that provide a plethora of ecosystem functions (e.g., Hesp 2002; Lewis 2005; Orth et al. 2006; Castillo et al. 2021). These functions result in ecosystem services essential to human society, such as carbon sequestration, water purification, and coastal safety (Costanza et al. 1997; Crossland et al. 2005; Martinez and Psuty 2008; Barbier et al. 2008; Brisson et al. 2014). Yet, anthropogenic pressures like development, exploitation, and eutrophication fuel ongoing habitat degradation, resulting in a growing need for conservation and restoration efforts worldwide (Bayraktarov et al. 2016; Fischman et al. 2019).

The sea-land interface is dominated by highly dynamic physical conditions generated through wind, waves, and tides, making it a harsh environment. Prevailing stressful environmental conditions exceed many species’ tolerances, especially in bare and unmodified areas (Crain and Bertness 2006; Balke et al. 2014; Castillo et al. 2021). Habitat-modifying seagrasses, marsh grasses, mangroves, and dune grasses may colonize these bare areas. Nevertheless, since early life stages are less stress tolerant than adults, establishment from seeds or plant fragments presents a bottleneck, and successful establishment or restoration requires two key components: (1) a period of favorable environmental conditions or ‘window of opportunity’, so seedlings and/or plant fragments may get rooted, thereby promoting growth and expansion (Balke et al. 2013, 2014; Altieri et al. 2013; Cao et al. 2018; Castillo et al. 2021; Lammers et al. 2024) and (2) sufficiently developed plant recruits to initiate self-facilitating biogeomorphic feedbacks that are often density or patch-size dependent (Corenblit et al. 2007; Bouma et al. 2009; Maxwell et al. 2017). Once plants intercept wind or water flow, sediment starts accumulating which in turn stimulates plant growth (Hesp 2002; Lewis 2005; Altieri et al. 2013; Möller et al. 2014). Subsequent environmental modifications ameliorate the prevailing physical stress, thereby facilitating the plant’s own vitality and clonal expansion (Altieri et al. 2013).

Although initially beneficial, ongoing habitat modification creates new ecological stressors over time. For instance, at first raised bed levels enable species to escape edaphic and inundation-related stress and may facilitate the growth of pioneer salt marsh grasses and mangroves (Bouma et al. 2005; Balke et al. 2016; Ellison et al. 2022). However, once bed level elevation reduces physical stress beyond species-specific thresholds, other less tolerant species could invade and outcompete the pioneers, marking the onset of succession (Bouma et al. 2005; Corenblit et al. 2015; Castillo et al. 2021; Ellison et al. 2022). Developing successional zonation across biogeomorphic stress gradients is characteristic of vegetated coastal systems, though it can vary (Crain and Bertness 2006; Corenblit et al. 2015; Torca et al. 2019; Castillo et al. 2021; Moreira-Saporiti et al. 2021). In seagrass, for instance, succession following initial colonization by pioneers is often absent or limited to a few species (Birch and Birch 1984; Moreira-Saporiti et al. 2021). Conversely, dunes feature distinct successional sequences expanding land inward (Doing 1985; Torca et al. 2019). Starting on the upper dry beach, burial-tolerant dune grasses trap and accumulate wind-blown sand, instigating embryo dune formation (Van Puijenbroek et al. 2017a, b). Ongoing burial stimulates the plant’s growth, further promoting sand accumulation and/or decreased erosion, enhancing dune height and stability, respectively (Maun 1998; Hesp 2002; Psuty 2008; Durán and Moore 2013). Via this amplifying sediment-growth feedback, the dune grass forms higher, more permanent foredunes to escape harmful seawater flooding (Huiskes 1979; Hesp 2002; Psuty 2008; Van Puijenbroek et al. 2017a). Over time, both foredune height and plant cover increase, mitigating ambient physical stresses (Hesp 2002; Psuty 2008; Durán and Moore 2013). Lacking fresh sand deposition, the dune grass starts to age as its sediment-growth feedback loop is obstructed (Marshall 1965). Simultaneously, the modified habitat facilitates the recruitment of less tolerant plants, paving the way for transitioning into stable, more diverse backdunes (Hesp 2002; Psuty 2008; Torca et al. 2019; Bonte et al. 2021). In essence, habitat-modifying plants alter their environment via feedback loops, generating biogeomorphic landscapes encompassing different stages of habitat modification. Initially beneficial, over time, landscape formation creates negative ramifications, restricting the spatial extent of habitat-modifying plants (Bouma et al. 2005). Yet, it enables succession allowing for vegetation and soil chronosequence, essentially underpinning species diversity across the biogeomorphic gradient in coastal landscapes (Crain and Bertness 2006; Corenblit et al. 2015).

**Fig. 1:**
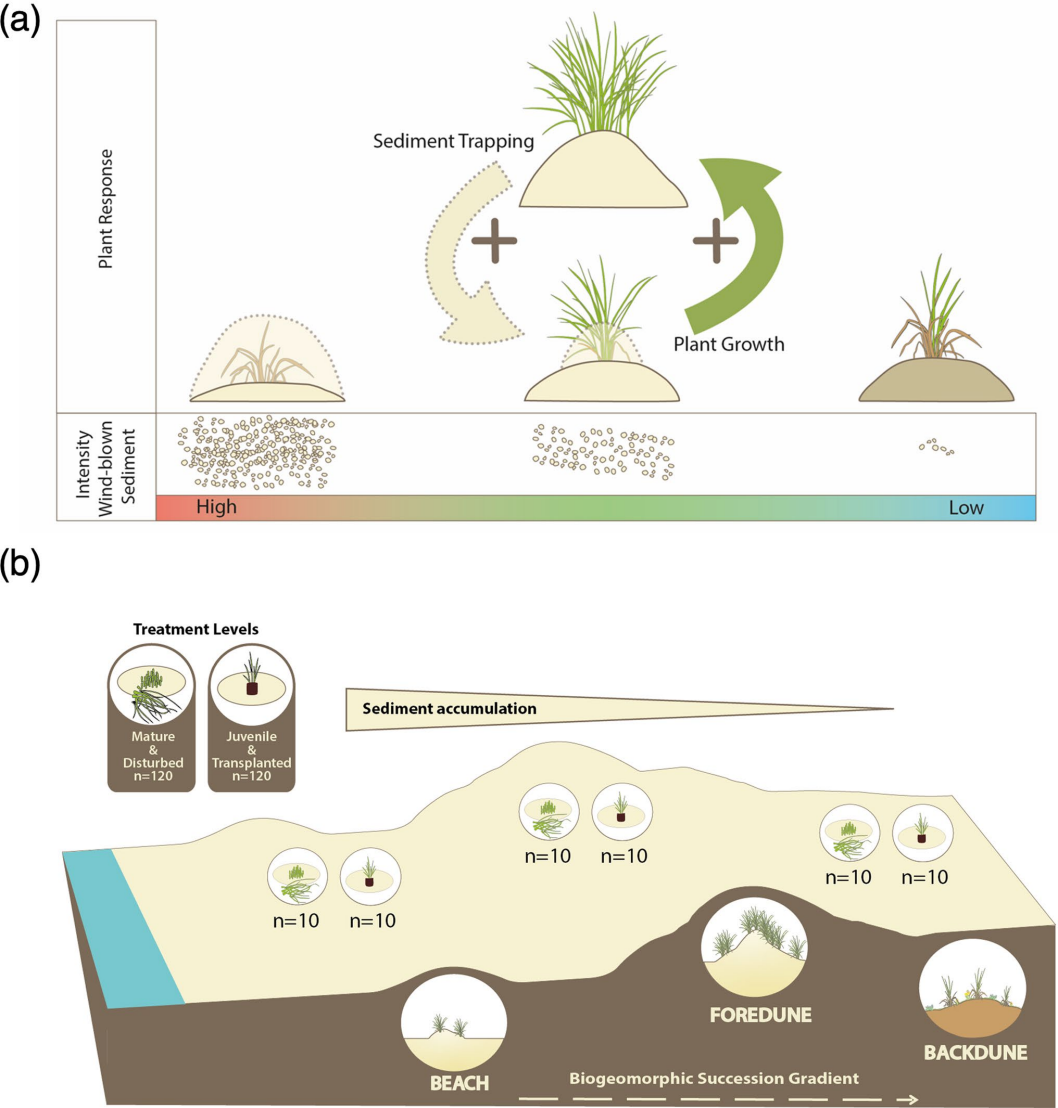
**a** Sediment-growth feedback mechanism **b** Visualization of the position of the three habitat types in relation to the biogeomorphological dune succession gradient and associated sedimentation stress

In Northwestern Europe, the above-described dune-building processes are dominated by European marram grass *Calamagrostis arenaria* (L.) Roth (Doing 1985), formerly *Ammophila arenaria* (L.) Link (hereafter marram grass). Marram grass’ burial tolerance, (a)biotic feedbacks, and dune building efficiency have been extensively studied in mature plants (e.g., Van der Putten et al. 1993; Maun 1998; Feagin et al. 2015; Bonte et al. 2021). Dominant across the early dune successional stages, marram grass disappears in the less stressful and more stable backdunes. Previous studies related this spatial constraint to changing stress gradients. In fact, prior work demonstrates that marram grass depends on burial to optimize growth and vitality (Van der Putten et al. 1990, 1993; Brown and Zinnert 2018; Nolet et al. 2018; Reijers et al. 2020; Ievinsh and Andersone-Ozola 2020). Under continuous deposition of wind-blown, mostly pathogen-free, beach sand, the newly forming roots escape soil pathogen infestation, while amplified vertical elongation allows re-surfacing, in turn promoting sand trapping. Yet, burial becomes lethal when exceeding marram grass’s vertical growth capacity, which may differ substantially between life stages (Reijers et al. 2020; Bonte et al. 2021; Lammers et al. 2024). Contrarily, negligible sand deposition enhances soil pathogen accumulation, previously linked to root damage invoked senescence and mortality (Fig. 1a). Similar to other habitat-modifying plants (e.g., Maxwell et al. 2017; Schwarz et al. 2018) the sediment-growth feedback, may therefore suddenly shift between amplifying or hampering marram grass vitality depending on both the sediment deposition rate and burial tolerance. This suggests that the benefits marram grass derives from habitat modification differ, both across the biogeomorphic dune succession gradient (hereafter referred to as dune gradient) and between life stages. Yet, to the best of our knowledge, no study has directly compared the response of different life stages of marram grass to altered sediment deposition rates across its full succession gradient.

In our study, we investigate how ongoing habitat modification—resembled by the position across the dune gradient—affects marram grass at two critical life stages (i) establishment of juvenile transplants and, (ii) resilience of mature plants to point-disturbance caused by above-ground biomass removal. We hypothesize the establishment success of juvenile transplants to be highest under mitigated but ongoing sediment deposition in the partly stabilized foredune, yet resilience in mature marram grass to decrease gradually with ongoing habitat modification and ceasing sediment deposition across the dune gradient. To test our hypothesis, we conducted a field experiment in three distinct dune habitats (i.e., the beach, the foredune, and the backdune) representative of consecutive successional modified zones along the dune gradient at four sites on the Dutch Wadden Island of Terschelling (Fig. S1). In a two-fold setup, we tested the establishment success of juvenile transplanted marram grass and the resilience of mature marram grass to above-ground biomass removal by point disturbances such as storm surges and grazing (Harris and Davy 1986; Feagin et al. 2015). To test establishment success, we used juvenile transplants from a nursery and planted them in close vicinity to the disturbed plants. Per treatment level, we monitored plant survival and morphology as metrics of establishment success and resilience over the first growing season [April to September 2021 (Huiskes 1979)].

## Methods

### Site description

We performed our field experiment between April and September 2021 in the dune system of the Dutch Wadden Island Terschelling, the Netherlands (53° 24′ N, 5° 21′ E). For the position across the dune gradient (further referred to as habitat), we distinguished between three distinct stages of habitat modification (beach, foredune, and backdune). The ‘beach’ encompassed the upper end of the beach, delineated by the presence of embryo dunes or the start of the foredune’s dune foot. This habitat is sparsely vegetated with extensive areas of bare sand. Marram grass co-occurs here alongside sand couch (*Elytrigia juncea*), the second dominant European beach grass (Fig. S1). We defined the ‘foredune’ as the area atop the first foredune ridge, characterized by denser vegetation cover dominated by marram grass (Fig. S2). The ‘backdune’, referred to the area behind the first foredune ridge showing evidence of succession. This zone featured a notable decline in marram grass cover in conjunction with the colonization of mosses, herbs, other grasses, and shrubs (Fig. S3). We selected four sites varying in their distances to sea per habitat, distance between the foredune and backdune, and foredune height (Fig. S4), as all these parameters can lead to differences in sediment deposition rate per habitat. The four sites were distributed over 13.5 km along the North Sea shore.

### Experimental setup

We performed a two-level multifactorial, paired-plot experiment initiated in April 2021. Per treatment level (i.e., establishment vs. disturbance) and at each site, we used 10 replicates per habitat for a total of 40 replicates for each habitat and treatment (*n* = 240) (Fig. 1b). For the disturbance treatment, we randomly selected 10 established, vital-looking marram grass tussocks per habitat and site, with a minimum distance of 2 m between adjacent plots. To minimize variability between experimental plots, we aimed for tussocks of similar size and vitality, growing in conditions representative of the local environment. To ensure marram grass tussocks were single-standing, we turned the soil to a depth of approximately 50 cm and in a radius of 50 cm around each plant. We further severed roots, as previous research found no negative ramifications for marram grass performance (Reijers et al. 2019), which we removed together with all other vegetation to exclude plant competition. We disturbed each plant by clipping and removing all above-ground biomass at ground level. The juvenile marram grass used in the establishment treatment was grown in potting soil and purchased from a commercial plant nursery (Kwekerij Zwartjes, NL), which also provides transplanted used by the Dutch government and local municipalities. We planted the 4-month-old juvenile transplants with their potting soil within a 10 m radius of the paired disturbed plot to minimize variability in the physical and biotic growth environment. To forestall possible effects caused by individual transplant fitness, we used 4 juvenile marram grass transplants per establishment plot (mean shoot count #17; mean shoot diameter 1.52 mm; mean longest shoot 52.4 cm). We selected a mostly vegetation-free area and further reduced biotic pressure by digging a hole with a radius of 50 cm and a depth of approximately 70 cm, turning around the soil and removing all vegetation and roots. Afterward, we filled the hole back up to ~ 10 cm depth (Willis 1965) and placed 4 juvenile transplants in a squared arrangement. We finished by burying the transplant with the previously excavated sediment up to surface level. To estimate local sediment stability, we inserted fiberglass rods (1 m × 3 mm) in the center of each plant to a fixed depth of 50 cm to track the relative change in bed level as a proxy for erosion and accretion dynamics and habitat modification per treatment level. To aid relocation during consecutive visits, we recorded the coordinates of each plot using an RTK GPS (TopCon). The experiment ran for one growing season, a period commonly used when assessing establishment and resilience in high-disturbance coastal environments (Van der Putten 1990; Reijers et al. 2019; Fischman et al. 2019).

### Data collection

We determined plant survival and vitality at the start and end of the experiment (April and September 2021) for both treatment levels based on three morphological parameters (shoot count, shoot diameter, maximum shoot length). A plant had survived the growing season when we were able to relocate it during the last measurement in September 2021, exhibiting at least one visible living shoot. Per plant, we counted all live shoots, measured the shoot diameter (mm) at the base of four randomly selected alive shoots, and the maximum shoot length (cm) from shoot base to highest leaf tip. All data was collected in the field, except for the starting vitality of the establishment treatment. Here, we collected data on 20 randomly selected transplants in the lab and used mean values during further analyses. We revisited all experimental plots six times throughout the growing season (161 days). During each visit, we determined the change in bed level (cm) inside each plant as an estimation for periodic erosion and accretion dynamics by measuring the above-ground fiberglass rod length (from tip to sediment surface level), after which we reset the rod to a standardized length.

### Statistical analysis

#### Plant survival and morphological development

The effect of habitat on the survival rate was tested by applying a Wilcox-signed rank test utilizing ‘Holm’ adjustment for multiple comparisons. To investigate the adult plant’s post-disturbance recovery and growth of the juvenile transplants, we calculated the relative change (RC) per plant parameter using the following formula (Reijers et al. 2019) (1):

1 $${\text{Relative}}\,{\text{Change }}\left( {{\text{RC}}} \right) = T_{{{\text{end}}}} /T_{{{\text{start}}}}$$

With *T*_start_ representing the initial measurements in April and *T*_end_ the final measurements after 161 days in September. A RC = 1 indicates no change, RC > 1 an increase, and RC < 1 a decrease of the plant parameter compared to the start. This method allowed us to compensate for differences in mature plant sizes at the start. We then created separate linear mixed-effect models for both treatment levels (establishment and disturbance), assessing the main effect of habitat on the three plant parameters’ RC using the site as a random effect. In two cases, site was not a significant random effect [establishment treatment—shoot count (RC), disturbance treatment—shoot length (RC)]. We subsequentially simplified these to linear fixed-effect models. For the mixed-effect models, we performed type III one-way ANOVAs (*α* > 0.05) with a Satterthwaite approximation of the degrees of freedom for each plant parameter and per treatment level. We used type II SS one-way ANOVAs (*α* > 0.05) for the two fixed-effect models. To separate habitat levels, we followed up significant ANOVA results with a Tukey HDS post-hoc test. The normality and homoscedasticity of the data were checked graphically, and we transformed the shoot count (RC) of the disturbance treatment utilizing Boxcox transformation (*λ* = − 0.061) to meet model assumptions.

#### Sediment stabilization and plant response

We calculated the standard deviation of the six bed-level-change (cm) measurements as a proxy for plot-level accretion-erosion dynamics. A large standard deviation, caused by large fluctuations in bed level change over time, indicates stronger accretion-erosion dynamics. Vice versa, a low standard deviation is representative of relatively stable conditions and weak accretion-erosion dynamics. We used the accretion-erosion dynamics (STDEV bed level change) to test for the effect of sediment stabilization by habitat modification across the biogeomorphological gradient on the juvenile establishment and mature resilience. For the juveniles, we tested the relationship between accretion-erosion dynamics (STDEV bed level change) and survival using a generalized linear model with binomial distribution and type II Chi-square one-way ANOVA (*α* > 0.05). Next, we determined if sediment stability differed across the biogeomorphological gradient in the disturbance treatment. Therefore, we performed a type II one-way ANOVA on a linear fixed effect model assessing the main effect of habitat on Boxcox transformed (*λ* = 0.101) accretion-erosion dynamics (STDEV bed level change). We separated the effect of habitat on accretion-erosion dynamics with a Tukey HSD post-hoc test. Subsequently, we tested the relation between the recovery of mature marram grass and local accretion-erosion dynamics via linear regression. For shoot count and diameter, we used linear mixed effect models with accretion-erosion dynamics (STDEV bed level change) as a fixed effect and site as a random effect. For the relation between shoot length (RC) and accretion-erosion dynamics (STDEV bed level change) we used a linear model, as the random effect of site was not significant. All model assumptions were graphically inspected. All statistical analyses were performed in R version 4.2.3 (15-Mar-2023). Summary statistics are presented as mean ± standard error (SE).

## Results

### Survival

**Fig. 2:**
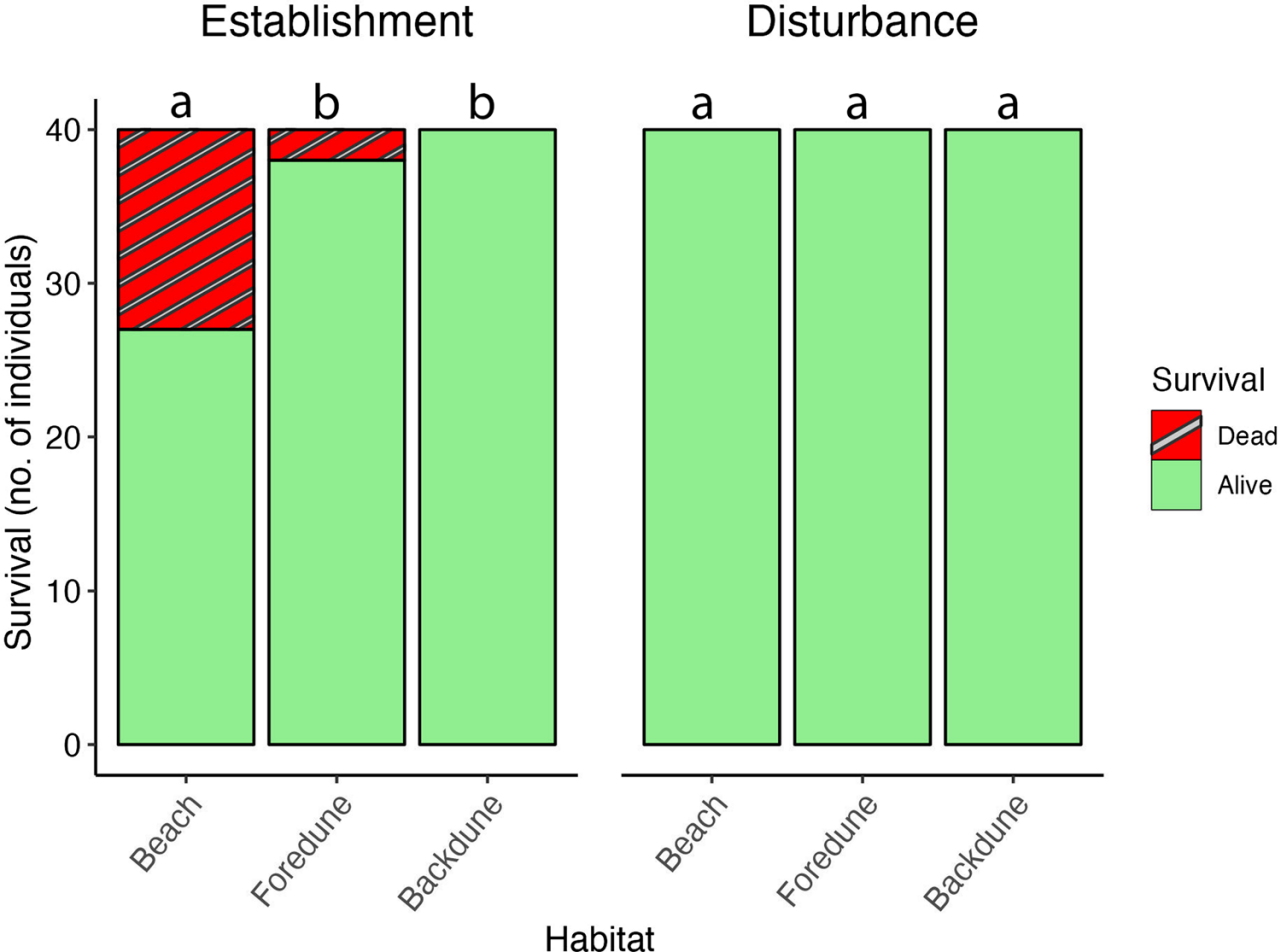
Count of alive and dead marram grass individuals at the end of the growing season in September 2021 (161 days) per habitat type for (left) establishment treatment, number of juvenile transplants, and (right) disturbance treatment, number of mature plants. Letters depict differences in Wilcox-signed rank (*P* < 0.05)

After 161 days, we saw a clear treatment-specific response in survival across the dune gradient. Out of the initial 120 juvenile transplants (*n* = 40 per habitat), 27 survived at the beach (67.5%), which is significantly lower than the 38 transplants (95%) in the foredune (Wilcox signed-rank test adjusted *P* = 0.004) and 40 transplants (100%) in the backdune (Wilcox signed-rank test adjusted *P* < 0.001), respectively (Fig. 2-Left). With two juvenile transplants dying in the foredune, the backdune was marginally but not statistically safer (Wilcoxon signed-rank test adjusted *P* = 0.16). Conversely, all 120 disturbed plants (100%) survived. This indicates that during the first season after above-ground biomass removal survival of mature marram grass is not affected by its position on the dune gradient (Fig. 2-Right).

### Recovery and plant morphological development

#### Habitat effect

**Fig. 3:**
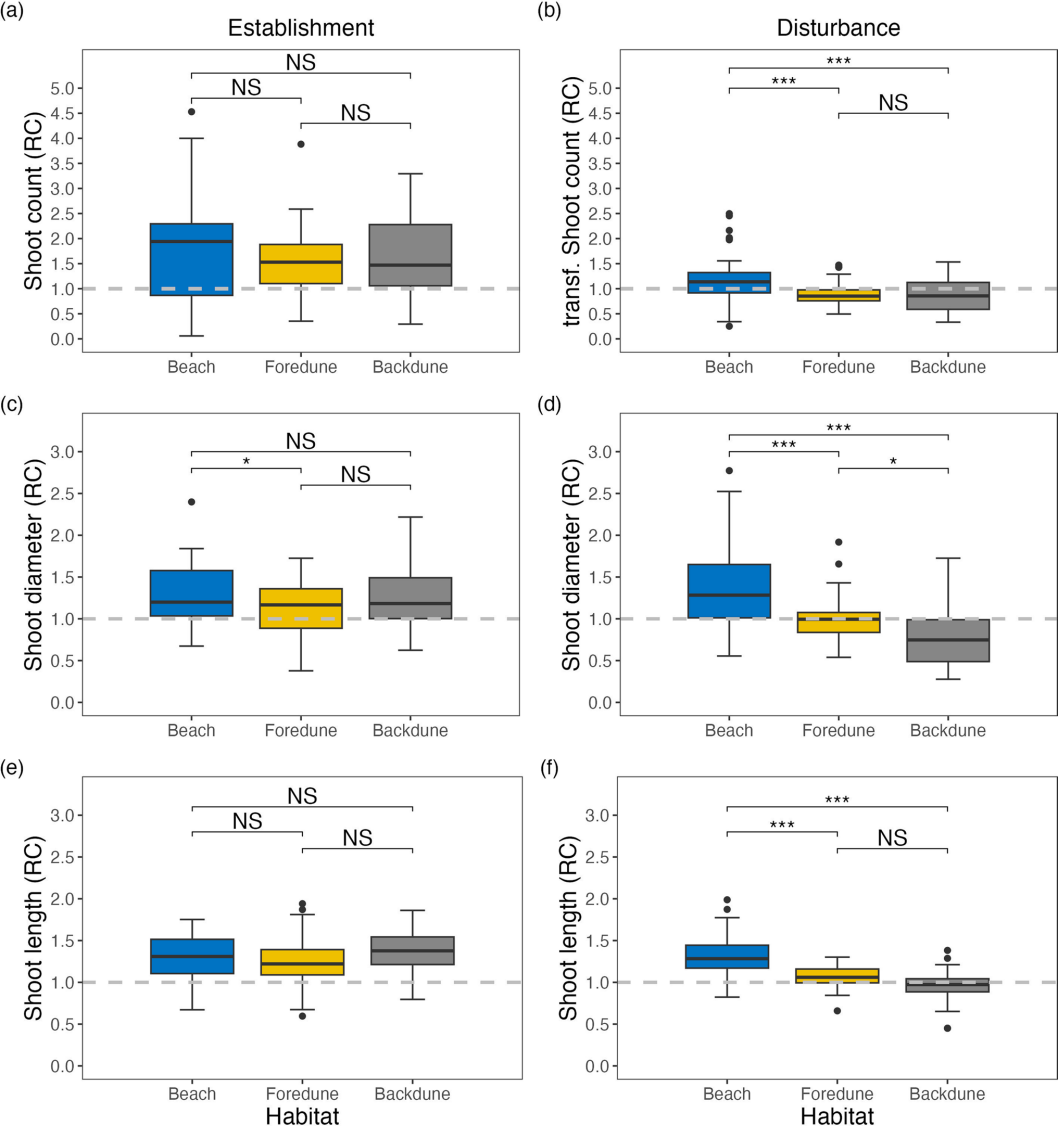
ANOVA results of the plant morphological development after a 161-day period relative to the starting condition presented as relative change (*T*_end_/*T*_start_) for the establishment treatment of juvenile transplants (**a**, **c**, **e**) and the disturbance treatment of the mature marram (**b**, **d**, **f**). The grey dotted line indicates a RC = 1 no change, above = increase, below = decrease. Top panels (**a**, **b**) illustrate the change in the number of alive shoots. Center panels (**c**, **d**) depict the change in average shoot diameter. Bottom panels (**e**, **f**) indicate the change in the maximum length of the live shoots. Significance codes: 0 ‘***’, 0.001 ‘**’, 0.01 ‘*’, 0.05 ‘.’, 0.1 ‘NS’

Next, we analyzed the effect of position (habitat) across the dune gradient on the plant morphological development for the establishment and disturbance treatment, respectively. We found that in the establishment treatment, the majority of the surviving transplants increased their shoot count, shoot diameter, and maximum shoot length over the growing season (relative change (RC) > 1) with little variation across the dune gradient (shoot count *F*_2, 101_ = 0.61, *P* = 0.55; diameter *F*_2, 99.05_ = 3.92, *P* = 0.023; length *F*_2, 99.36_ = 2.00, *P* = 0.14) (Fig. 3a, c, e). The only exemption is the shoot diameter, where juvenile transplants on the beach grew significantly thicker stems than the ones in the foredunes (Fig. 3c).

In the disturbance treatment, plants did not necessarily recover to pre-disturbance states in each habitat (shoot count *F*_2,112.02_ = 12.95, *P* < 0.001, diameter *F*_2, 111.02_ = 31.69, *P* < 0.001, length *F*_2,115_ = 34.34, *P* < 0.001). Marram grass growing on the beach had on average more shoots with thicker and taller stems in September compared to the start of our experiment in April (RC > 1). In contrast, plants in the foredune mostly failed to reach pre-disturbance shoot count (RC < 1). However, the shoots that recovered reached approximately the same stem thickness as prior to disturbance (RC = 1), while overall increasing their shoot length compared to their starting conditions (RC > 1). Lastly, the backdune plants had on average fewer shoots in September, and those were mostly smaller and thinner compared to pre-disturbance in April (RC < 1) (Fig. 3b, d, f). Overall, mature marram grass recovered significantly better on the beach than in the foredune and backdune (shoot count, diameter, length Tukey *P* < 0.001, respectively), while plants in the foredune performed similarly to the backdune only growing thicker shoots (diameter Tukey *P* < 0.01).

#### Sediment stabilization and plant response

**Fig. 4:**
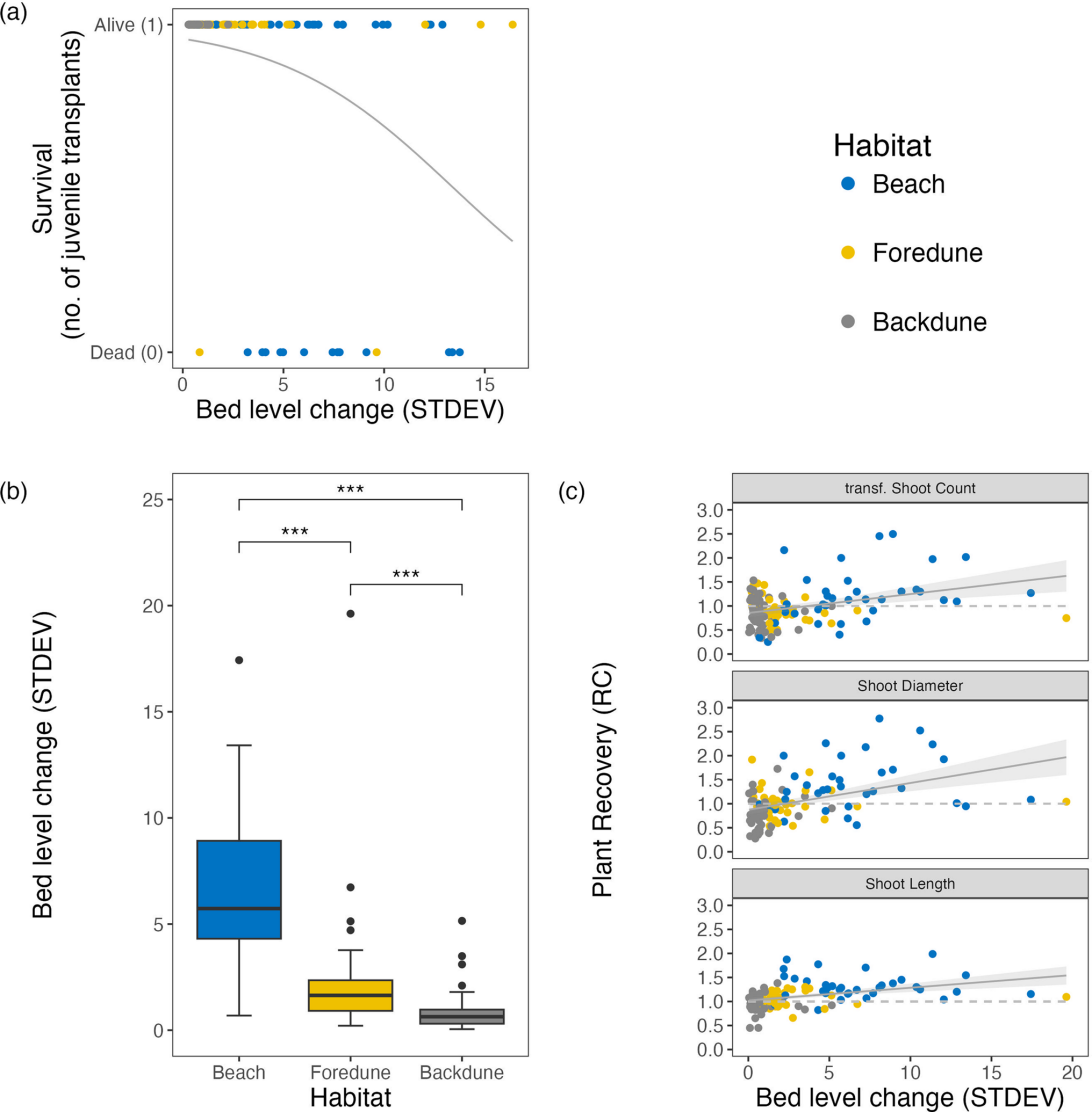
**a** Establishment treatment: binomial regression on the survival of the juvenile transplants in relation to bed level change (STDEV) (Df = 1, Chisq *P* < 0.001). **b** Disturbance treatment: ANOVA on the differences in the variation in sediment accumulation at the disturbance plants measured as the standard deviation of bed level change (cm) over the six-time points per habitat. **c** (top) relation between the transformed shoot count recovery (RC) rate and bed level change (STDEV) (marginal *r*2 = 0.09 and conditional *r*2 = 0.319, *P* < 0.001); **c** (middle) relation between diameter recovery rate (RC) and bed level change (STDEV) (marginal *r*2 = 0.168 and conditional *r*2 = 0.406, *P* < 0.001); **c** (bottom) relation between the maximum shoot length recovery rate (RC) and bed level change (STDEV) (multiple *r*2 = 0.161 and adjusted *r*2 = 0.153, *P* < 0.001). Significance codes: 0 ‘***’, 0.001 ‘**’, 0.01 ‘*’, 0.05 ‘.’, 0.1 ‘NS’

Lastly, we assessed how far the above-described responses of juvenile transplanted and mature disturbed marram grass across the dune gradient related to local accretion-erosion dynamics (STDEV bed level change). We found that for the juvenile transplants, low survival chances at the beach significantly relate to high local accretion-erosion dynamics (GLM, *P* < 0.001) (Fig. 4a). Conversely, the significant decrease in accretion-erosion dynamics across the dune gradient (STDEV bed level change beach 6.57 ± 0.63 cm, foredune 2.30 ± 0.5 cm, backdune 1.01 cm ± 0.16 cm, Tukey, *P* < 0.001, Fig. 4b), negatively affected the recovery in the disturbance treatment. Larger differences in sediment deposition and erosion events at the beach (Fig. S5a) related positively to marram grass recovery. This effect disappeared in the similar but much more stabilized accretion-erosion dynamics in the foredune (Fig. S5b), and in the very stable partly erosive backdune lacking sediment input (Figs. 4c, S5c).

## Discussion

In this study, we examined how ongoing habitat modification—resembled by the position across the biogeomorphic dune succession gradient (beach, foredune, backdune)—affects the performance of the dune-building marram grass over one growing season at two critical life stages: (i) establishment of juvenile transplants and (ii) resilience to point-disturbance of mature plants. We found that the two life stages responded with opposing patterns to increasing sediment stabilization across the dune gradient. Where the stronger accretion-erosion dynamics at the beach, entailing both periodic deposition and erosion, hampered the survival of juveniles, it facilitated the recovery of mature plants. Contrastingly, ceasing accretion-erosion dynamics, mostly associated with net erosion in the backdune, did not affect the juvenile’s growth but impeded the recovery rate of mature marram grass.

For the establishment treatment, we found that the greater differences in both sediment deposition and erosion events at the beach, characteristic of a barely modified habitat, impeded the survival chances of juvenile transplants. Whereas with the onset of landscape formation ceasing accretion-erosion dynamics in the foredune was more favorable (Fischman et al. 2019). Yet, contrary to our expectations, the highly modified back dune lacking sediment deposition was most advantageous for transplant establishment. The benefits of low burial stress on establishment were likely further enhanced by the fact that we reduced competitive stress by removing the surrounding vegetation at the start of the experiment. Contrastingly, all mature marram grass exposed to the disturbance treatment survived the first growing season. The observed survival rate at the unmodified, highly dynamic beach and partly modified semi-dynamic foredunes coincides with earlier findings (Reijers et al. 2019). Unexpectedly, the lack of sediment deposition in the highly modified, mostly stable and partly erosive back dune conditions in our experiment did not lower post-disturbance survival chance. While such heavily modified conditions allow for the accumulation of harmful soil microbes and increasing successional pressure, it appears that these degenerating forces associated with the demise of marram grass (Van der Putten et al. 1988, 1990; McLachlan 1991; Van Der Stoel et al. 2002; Torca et al. 2019; Bonte et al. 2021) were not severe enough to negatively affect the survival of disturbed plants within one growing season. The lower survival chances of the juveniles under more dynamic accretion-erosion conditions suggest that their burial tolerance is inferior to disturbed mature marram grass. This difference likely arises from the larger below-ground network of mature plants, enabling them to substitute photosynthetic energy under complete burial, otherwise deemed fatal (Maun et al. 1996; Maun 1998). Our results suggest that the role of accretion-erosion dynamics in determining marram grass survival shifts across its life cycle. Initially, being a key limiting factor during juvenile establishment, it becomes indifferent to the post-disturbance survival of mature plants.

Surprisingly and contrary to earlier studies (e.g., Fischman et al. 2019; Ievinsh and Andersone-Ozola 2020; Reijers et al. 2020), juveniles grew similarly well across the dune gradient in our experiment. This suggests that growth stimulation by sediment deposition is not essential for juvenile transplants in their first growing season. However, we only considered above-ground biomass, potentially masking sedimentation-driven variation in resource allocation and expansion of below-ground biomass (e.g., Fischman et al. 2019; Ievinsh and Andersone-Ozola 2020; Reijers et al. 2020). As expected and contrary to survival, the growth of the disturbed marram grass significantly decreased with progressing habitat modification across the dune gradient. The unmodified, dynamic conditions at the beach created by fluctuating sediment deposition and erosion events allowed marram grass to even exceed pre-disturbance morphological characteristics. Yet with lacking sediment input, and small erosion events in the highly modified, stable backdunes marram grass mostly failed to recover (Van der Putten and Peters 1995). Although differences in recovery potential between beach and foredune have been attributed to reduced nutrient input (Reijers et al. 2019), it is still unclear what exactly causes the vitality of marram grass to diminish with ceasing sediment input (De Rooij-van Der Goes et al. 1995; Bonte et al. 2021). It is beyond the scope of this study to disentangle all underlying forces, but in line with earlier work (e.g., Van der Putten and Peters 1995), we can link poorer plant performances across the dune gradient to ceasing accretion-erosion dynamics due to a lack of fresh sediment input caused by landscape formation. This suggests that obstructing sediment-growth feedbacks and associated negative effects (e.g., Van der Putten and Peters 1995; Bonte et al. 2021) are inhibiting the recovery potential of mature marram grass.

Although we cannot directly translate our findings to marram grass’ naturally occurring population dynamics since we used nursery-grown transplants, our work supports the notion that the environmental niche of marram grass changes across its life cycle (Del Vecchio et al. 2020). On the one hand, strong accretion-erosion dynamics characteristic of unmodified beach and embryo dune significantly limit the establishment of juvenile transplants. On the other hand, the same dynamic conditions significantly improve the resilience of mature established marram grass. Overall, this suggests that the sediment-growth feedback loop of marram grass and associated habitat modification and landscape formation creates a trade-off negatively affecting the establishment chances of juveniles while enhancing mature plant resilience.

The existence of feedback-induced trade-offs in coastal habitat-modifying plants has implications for both plant population dynamics and landscape formation. For instance, self-facilitation via feedback loops requires a dynamic physical environment, which may hamper establishment success. By restricting establishment to a ‘window of opportunity’, feedback loops affect population dynamics (e.g., Balke et al. 2013; Silliman et al. 2015; Maxwell et al. 2017; Cao et al. 2018; Fischman et al. 2019; Steinigeweg et al. 2023). Moreover, plant-specific traits determine the engineering capacity, in turn controlling the type of landscape formed, which feeds back into habitat diversity (e.g., Bouma et al. 2005; Peralta et al. 2006; Schwarz et al. 2018). In salt marshes, for example, *Spartina spp.* (cordgrass) builds high tussocks as its stiff stems maximize sand trapping, but that comes at the cost of increased vulnerability to hydrodynamic stress (Bouma et al. 2005; Van De Ven et al. 2023). While high tussocks locally reduce stress, thus facilitating survival, the resulting elevation gradient increases flow velocity at tussock edges thus enhancing marsh erosion and cliff formation (Cao et al. 2021). Moreover, scouring and hydrodynamic stress at the edges further limits clonal expansion (Van Hulzen et al. 2007; Van De Ven et al. 2023). Such phenomena, where habitat modification creates opposing so-called ‘scale-dependent’ feedbacks, are key agents in landscape formation (Van Wesenbeeck et al. 2008). In other words, habitat-modifying plants face a trade-off between overcoming patch- and density thresholds for local facilitation and larger-scale inhibition by increasing stress due to their vicinity. Thus, for coastal vegetated systems to persist in a dynamic state, there must be a balance between trade-offs enabling the establishment, growth, and resilience of habitat-modifying plants. Unfortunately, ongoing habitat degradation, e.g., infrastructure-induced changes in sedimentation, interfere with these biogeomorphic feedbacks, fueling the need to enhance our knowledge about species- and life stage-specific requirements of habitat-modifying plants for habitat restoration and conservation (Lewis 2005; Maxwell et al. 2017; Schwarz et al. 2018; Van Hespen et al. 2022).

For marram grass, high burial tolerance and even dependency on the sediment-growth feedback to sustain vitality are well documented (e.g., Van Der Stoel et al. 2002; Zarnetske et al. 2012). However, here we demonstrate that the effects of the sediment-growth feedback are life-stage dependent. While sediment stabilization in heavily modified backdunes benefits juvenile establishment, mature marram grass requires high sediment dynamics found in less modified beach and dune environments for recovery. Our finding ties in with recent work demonstrating that on a small scale, habitat modification by adult marram grass has a direct negative effect on seedling emergence (Lammers et al. 2024). Thus, like in other habitat-modifying plants, feedback-driven landscape formation generates trade-offs between marram grass’s life stages. Hence, it is pivotal to understand how environmental requirements change throughout the life cycle of habitat-modifying plants and to assess how biogeomorphic feedbacks and landscape formation influence plant performance (Grubb 1977; Schwarz et al. 2015; Fischman et al. 2019; Del Vecchio et al. 2020; Török et al. 2020).

Acknowledging life-stage dependent variation in environmental requirements of habitat-modifying species has direct implications for restoration and conservation efforts. For instance, marram grass transplants are still commonly used in dune restoration projects. Recently, the suitability of traditional transplanting strategies for restoration success has been questioned (Fischman et al. 2019). One possible way to enhance restoration could be to select sites based on local stress gradients (Fischman et al. 2019), with higher sediment dynamics favorable for the growth and resilience of mature plants and facilitate transplant establishment by placing temporary structures e.g., windscreens. For conservation purposes like improving the coastal resilience of more mature dunes, we emphasize that reinstating sedimentation dynamics by removing vegetation or by creating notches can enhance the vegetation recovery potential. Nevertheless, since our conclusions are based on a single growing season it remains open for how long the here-described trade-off between the safe establishment of juvenile- and the resilience of mature marram grass will last throughout the life cycle of marram grass.

Considering the key role habitat-forming plants like marram grass play in biogeomorphic coastal systems, our results underpin the importance of understanding life-stage-dependent differences in environmental requirements throughout a plant’s full life cycle. Therefore, to expand our understanding of marram grass performance beyond initial establishment and recovery success, experiments extending beyond a single growing season are required to predict its contribution to dune dynamics.

## Supplementary Information

Below is the link to the electronic supplementary material.

Supplementary file1 (DOCX 24207 KB)

## Data Availability

The data supporting this study are publicly available from the DataverseNL repository 10.34894/LCM4MF.
