# Supplementary material for: Two sides of the coin: Feedback-driven landscape formation results in trade-off between establishment and resilience of marram grass
Source: Oecologia. 2025 Apr 5;207(4):63. doi: 10.1007/s00442-025-05693-5 (PMC11972180; doi:10.1007/s00442-025-05693-5)
Supplement: Supplementary file 1 — Supplementary file1 (DOCX 24207 KB) [file 442_2025_5693_MOESM1_ESM.docx]

**Supplementary Material**

Two sides of the coin: Feedback-driven landscape formation results in trade-off between establishment and resilience of marram grass

Solveig Höfer^1,2^, Lisse de Groot^3^, Nathan Scanlan^2,4^, Eva Lansu^1,2^, Max Rietkerk^5^, Martin Wassen^5^, Tjisse van der Heide^1,2^, Valérie C. Reijers ^4^

**Corresponding author**

Solveig Höfer, [solveig.hofer@nioz.nl](mailto:solveig.hofer@nioz.nl), [solveig.hoefer@gmx.de](mailto:solveig.hoefer@gmx.de)

Tel: +31 222 369 575/ +31 6 225 521 85

**Data availability**

The data supporting this study are publicly available from the DataverseNL repository [https://doi.org/10.34894/LCM4MF](https://eur01.safelinks.protection.outlook.com/?url=https%3A%2F%2Fdoi.org%2F10.34894%2FLCM4MF&data=05%7C02%7C%7C5b172bc7195f4919acae08dd50dcfec5%7C9a1651bf58af435b86a83e9334b4b732%7C0%7C0%7C638755632313000820%7CUnknown%7CTWFpbGZsb3d8eyJFbXB0eU1hcGkiOnRydWUsIlYiOiIwLjAuMDAwMCIsIlAiOiJXaW4zMiIsIkFOIjoiTWFpbCIsIldUIjoyfQ%3D%3D%7C0%7C%7C%7C&sdata=zoaLulrolXkSCeYx3qHxRs6rDl2OOwLlwaiCP6dqzxE%3D&reserved=0)


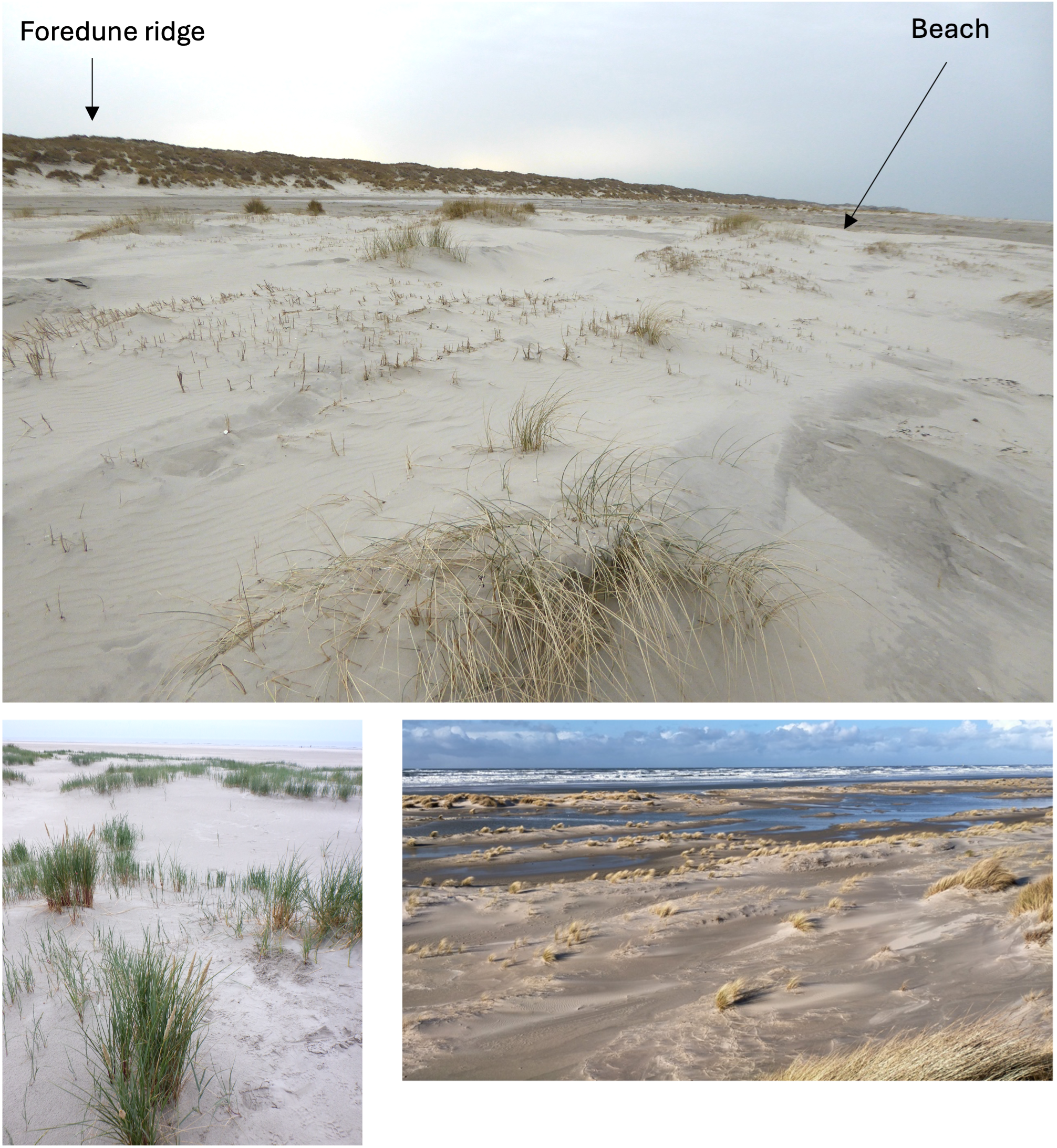


Fig. S1 Examples of the habitat type ‘beach’


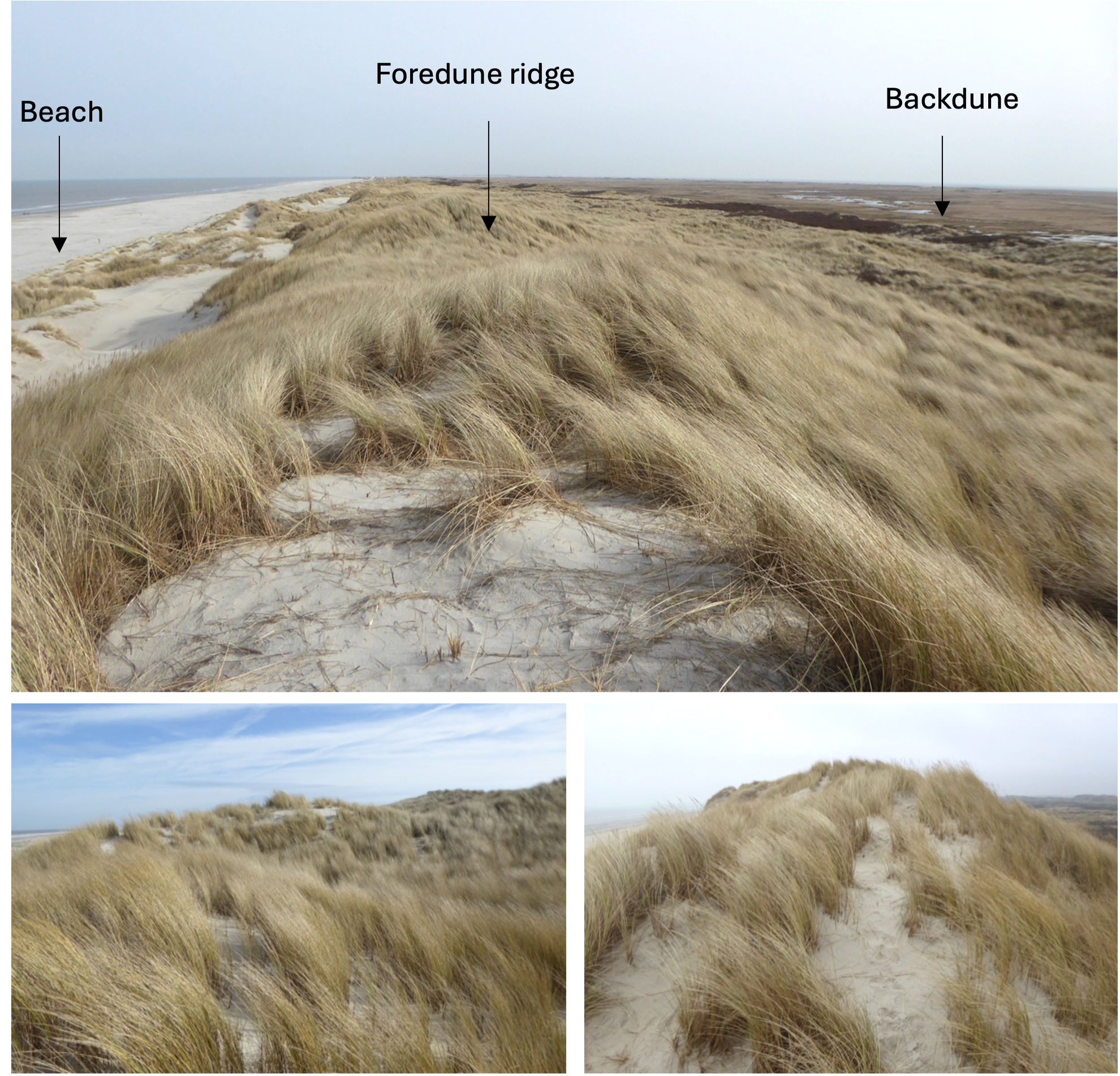


Fig. S2 Examples of the habitat type ‘foredune’


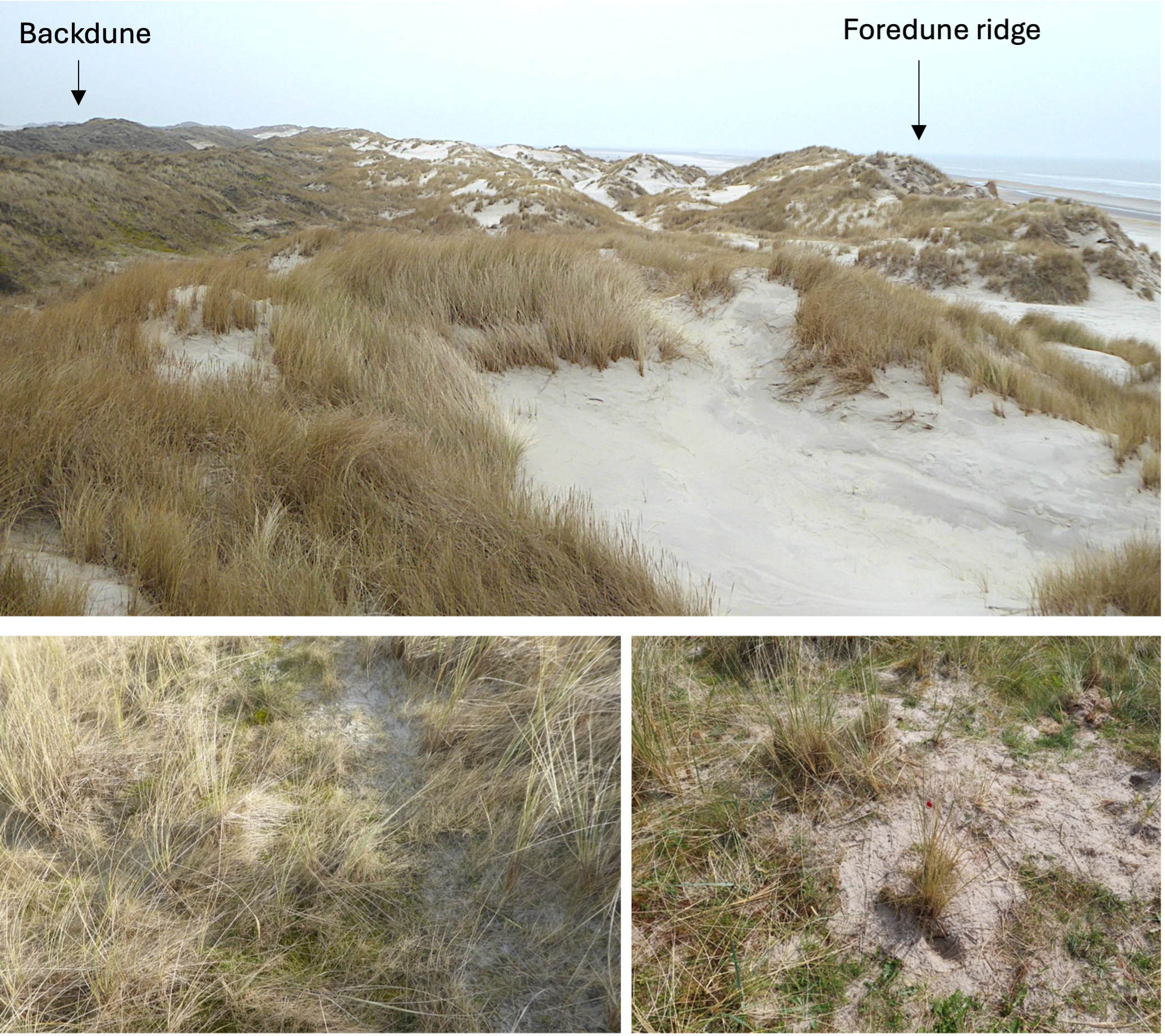


Fig. S3 Examples of the habitat type ‘backdune’


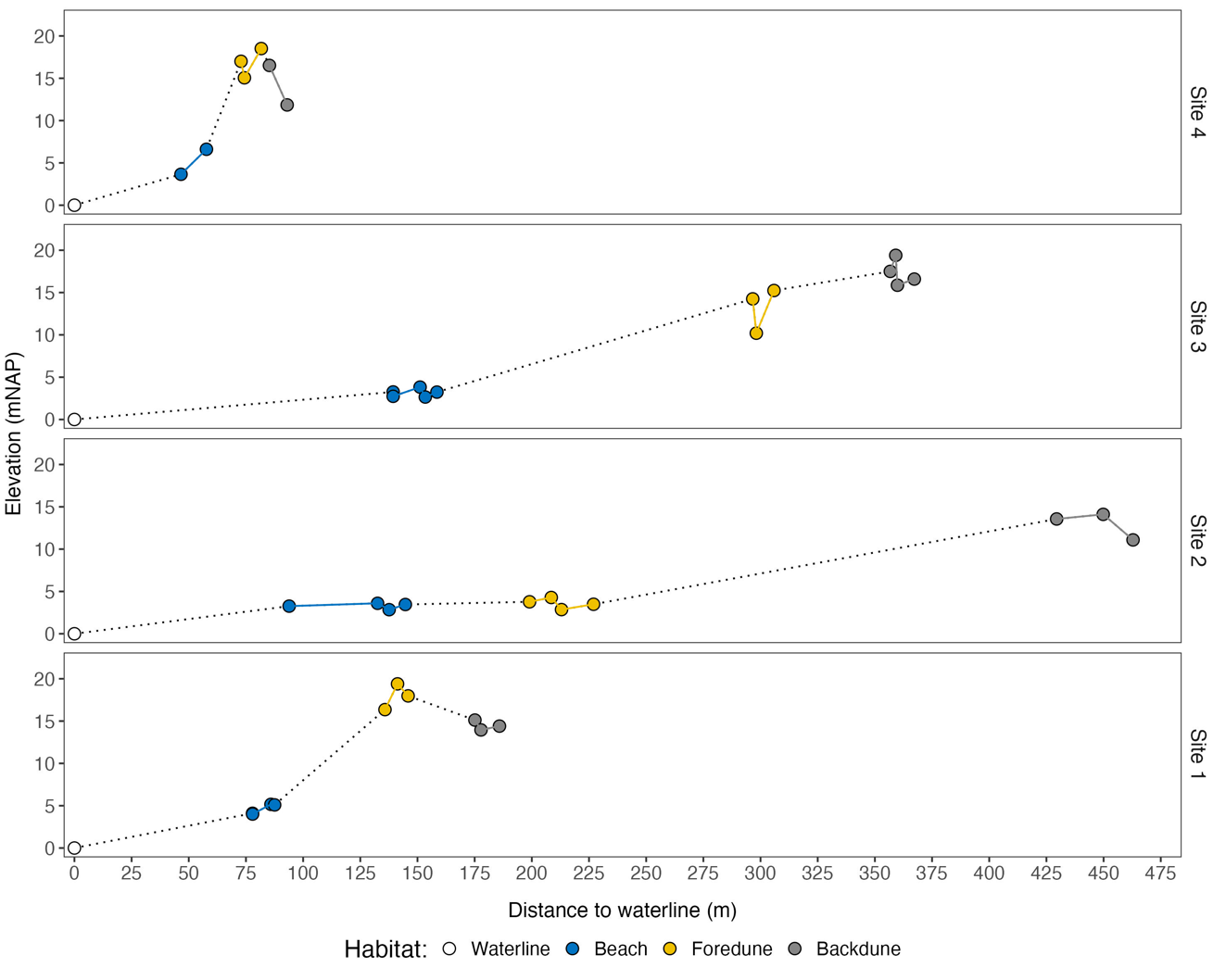

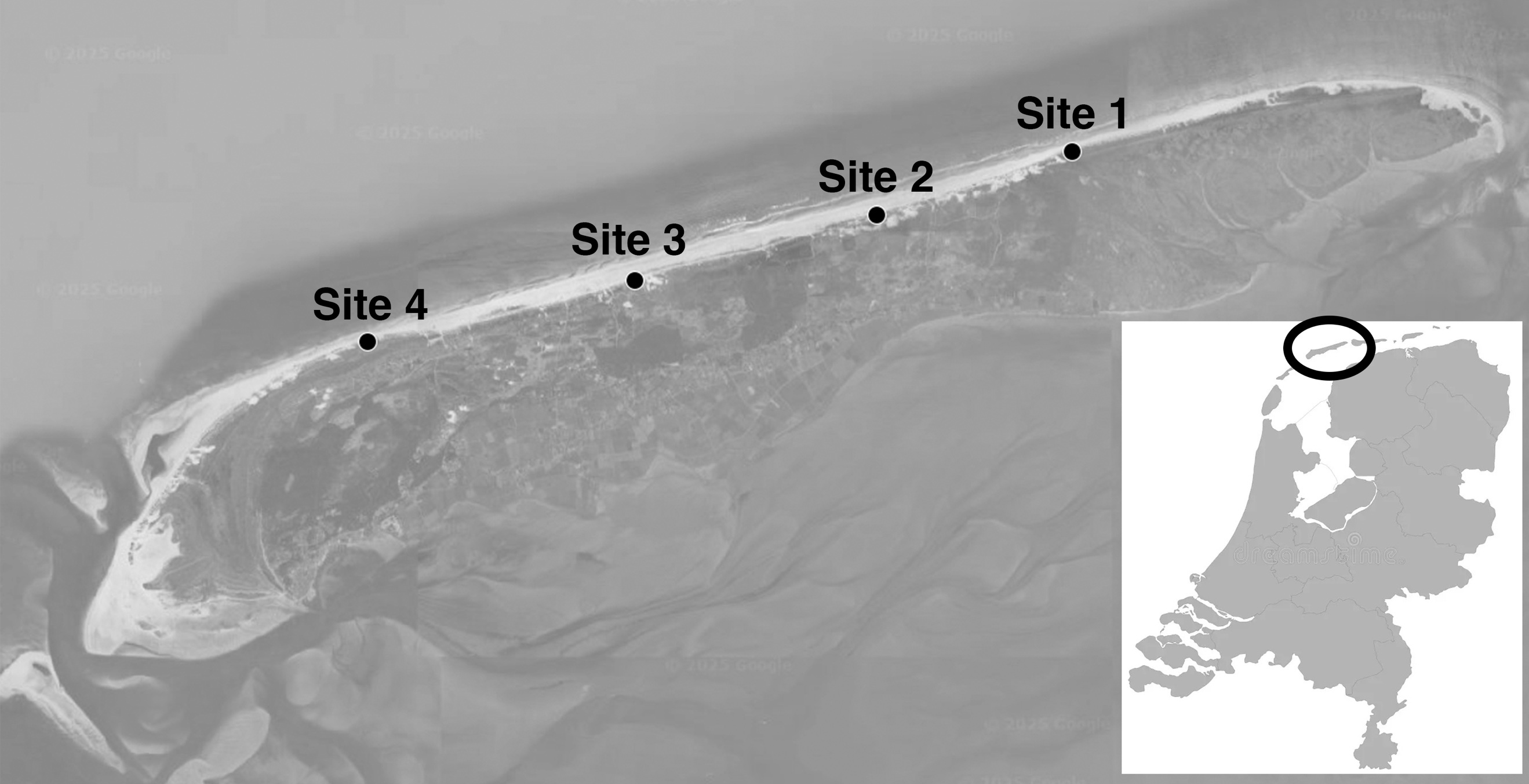


Fig. S4 The location of the four study sites on the Dutch Wadden island of Terschelling and the average distance to sea (m) and elevation (m) of the three habitat types per site


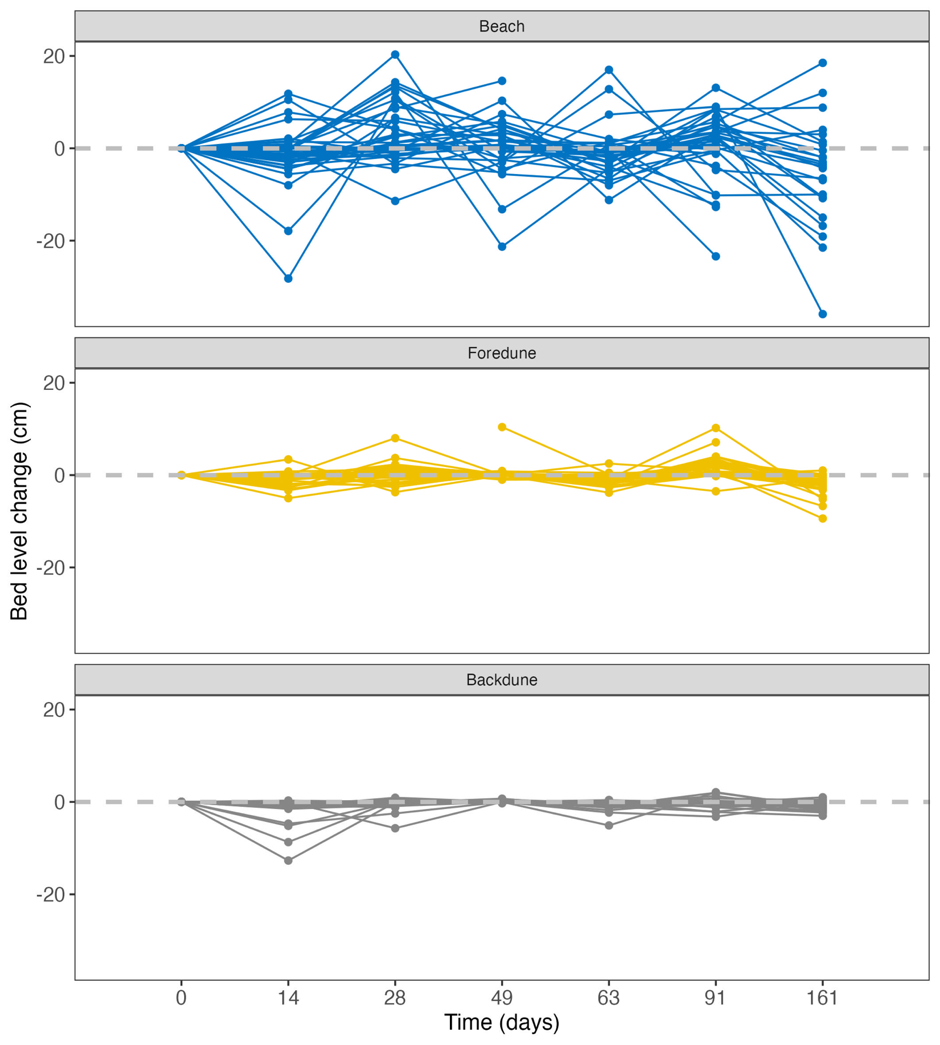


(a)

(b)

(c)

Fig. S5 Accretion-erosion dynamics, measured as the change in bed level (cm) in the center of each plot, over time starting in April 2021 (0) to the end is September 2021 (after 161 days) for the disturbance treatment plots at the beach (a), the foredune (b), and the backdune (c)
